# Cas9 immunity creates challenges for CRISPR gene editing therapies

**DOI:** 10.1038/s41467-018-05843-9

**Published:** 2018-08-29

**Authors:** Julie M. Crudele, Jeffrey S. Chamberlain

**Affiliations:** 1 Department of Neurology, University of Washington, Seattle, WA 98195-7720 USA; 2 Senator Paul D. Wellstone Muscular Dystrophy Specialized Research Center, University of Washington, Seattle, WA 98195-7720 USA

## Abstract

Clustered regularly interspaced short palindromic repeats (CRISPR)-Cas9 is a genome-editing technology^1,2^ that utilizes archaeal and bacterial Cas9 nucleases to introduce double-stranded breaks in DNA at targeted sites. These breaks can be used to remove, replace, or add pieces of DNA. While not the first genome editor, CRISPR-Cas9 is efficient and cost-effective because cutting is guided by a strand of RNA rather than a protein. The potential uses in health care are plentiful, from disrupting dominant genes that cause cancer^3^ to repairing mutated genes that cause genetic diseases, such as muscular dystrophy^4^. Therapeutic approaches based on this technology fill the preclinical pipeline, and rely on the use of viral vectors to deliver the *Cas9* gene and guide RNA to a gene of interest. However, concerns regarding the safety and efficacy of CRISPR-Cas9 use in gene therapy remain. A pre-print released prior to peer review has recently underlined the question of whether immunological responses to Cas9 may negatively impact its clinical use^5^. Here we discuss the implications of this finding for the application of CRISPR/Cas in gene therapy.

## Immunity against *Staphylococcus aureus*, *Streptococcus pyogenes*, and Cas9

The two most commonly used sources of Cas9 are *Staphylococcus aureus* (*S. aureus*; SaCas9) and *Streptococcus pyogenes* (*S. pyogenes*; SpCas9), which frequently colonize humans and cause disease (e.g. MRSA and strep throat). Both humoral, antibody-mediated, and cellular, T-cell-mediated immunity against these bacteria have been detected in more than 80% of healthy individuals^6–8^. However, the dominant responses are against secreted and surface proteins, to which the immune system has easy access to. So, since Cas9 is an intracellular protein, what does this mean for Cas9-mediated gene editing?

In a pre-print deposited in Biorxiv before peer review, Charlesworth et al.^5^ demonstrate that anti-Cas9 responses are present in healthy human adults. Of 34 blood samples probed, anti-Cas9 IgG antibodies were detected against SaCas9 in 79% of samples, and against SpCas9 in 65% of samples. Thirteen additional blood samples tested for anti-Cas9 T cells showed cellular immunity at rates of 46% to SaCas9 but 0% to SpCas9. However, the authors acknowledge that their system for detecting anti-Cas9 T cells is not as sensitive as other techniques and that T-cell responses to SpCas9 may be present at levels below the limit of detection.

While the existence of anti-Cas9 antibodies indicates that the immune system has been exposed to intracellular bacterial proteins during infection, they may not be relevant as anti-gene therapy immune responses. Antibodies are important for coating bacteria and viruses to block their entry into cells and to mark them for destruction by the immune system. They can also mark infected or cancerous host cells that express the target protein on their cell surface. Generally, however, antibodies against an intracellular protein will not directly lead to killing of a cell expressing that protein. Rather, killing is mediated through cellular immune responses—specifically, CD8+ cytotoxic T lymphocytes (CTLs)—not antibodies. It is therefore the rate of cellular immunity to Cas9 that is worthy of the most consideration. The existence of anti-Cas9 T cells in these donors^5^ indicates, first, that there are T cells that can react to Cas9 in the circulation and, second, that these T cells are being presented Cas9 effectively through major histocompatibility complex (MHC) molecules. Activation of these T cells with concomitant proinflammatory “danger” signals during a bacterial infection generates CTLs that can destroy infected host cells. While not directly tested in a killing assay, the Cas9-reactive CD8+ T cells detected by Charlesworth et al.^5^ do secrete interferon-γ, suggesting they could kill Cas9-expressing cells following gene therapy. In other words, the immune system may destroy the very cells CRISPR-Cas9 corrected, rendering the treatment useless.

## Implications for gene therapy and mitigating strategies

The threat of a CTL response against Cas9 and its implications for gene therapy depend on the context of editing. In ex vivo gene therapy, in which cells are treated in a dish before transplantation, Cas9 immune responses can potentially be circumvented by using transient Cas9 expression and waiting for the Cas9 protein to clear before administering the corrected cells to patients. Direct editing of cells in vivo, however, typically utilizes a viral-derived vector to deliver the *Cas9* gene, leading to long-term expression in the presence of an intact immune system, which could potentially trigger an immune response to Cas9.

For patients without anti-Cas9 memory T cells, the question remains as to whether gene therapy alone could elicit anti-Cas9 CTLs. Several factors determine whether there will be an immune response to a gene product following gene therapy: the inflammatory nature of the vector, the dose, and route of administration; the tissue specificity of the promoter; the target tissue; the underlying level of inflammation; and the gene product itself^9^. A recent article found proliferation of anti-Cas9 T cells following adeno-associated viral vector (AAV) intramuscular delivery of a split SpCas9 expressed from a ubiquitously active promoter^10^. However, there was no evidence that the resulting T cells were capable of killing. Rather, they were naive and immature T cells, which have also been seen with other AAV-delivered genes where there was immune-cell infiltrate but no destruction of the transduced tissue^11,12^. This was in contrast with evidence in mice where Cas9 was delivered through electroporation of naked DNA, leading to a destructive immune response^10^. Together, these data suggest that Cas9 itself is not necessarily a strong immunogen, and the context in which it is presented will determine the nature of the response. However, additional preclinical assessments of Cas9 immunogenicity should be performed in large animals known to model human immune responses to gene therapies, such as dogs and nonhuman primates, using clinical-grade vectors targeted to neither tolerogenic nor immune privileged tissues, with months of follow-up to more carefully assess the immunogenicity of various Cas9 proteins. Should a limited number of reactive epitopes be found—as was the case in Chew et al.^10^, which identified just one—these epitopes could be masked through mutation to prevent MHC binding and/or T-cell recognition.

To minimize the chance of developing anti-Cas9 CTLs when performing CRISPR-*Cas9* gene therapy, known strategies should be employed. To lessen the risk of an immune response, first-in-human trials should perhaps be in immune-privileged (e.g. eye) or tolerogenic (e.g. liver) organs while the immunogenicity of Cas9 in humans absent a concomitant bacterial infection is assessed. Special care should also be taken during treatment of tissues with underlying inflammatory diseases, as pro-inflammatory environments may make the development of anti-Cas9 CTLs more likely. Less inflammatory vectors such as AAV, intravascular over intramuscular injections, the lowest efficacious doses for non-liver tissues or tolerogenic doses for liver, and tissue specific promoters that prevent expression in antigen-presenting cells should be chosen whenever possible. For example, a recent study used CRISPR-Cas9 to correct muscular dystrophy expressed Cas9 from a muscle-restricted regulatory cassette (*CK8*) following intravascular delivery with AAV^4^. Mice showed physiologic improvement even 18 weeks later, past the window of an expected immune response. *CK*-based promoters have also been shown to prevent a CTL response against *Escherichia. coli* β-galactosidase^13^ and are currently being used in several AAV clinical trials for muscular dystrophy.

Preventing the immune destruction of CRISPR-Cas9-corrected cells could be more challenging in patients that already have pre-existing anti-Cas9 CTLs, since preventing the differentiation and activation of new CTLs is easier than inhibiting those already in existence. If inflammation is minimized during gene delivery, expression of Cas9 sans “danger” signals could lead to anergic, nonresponsive T cells. Unfortunately, inflammation could result in expansion of CTLs and killing of treated cells. Depending on the inflammatory nature of the therapy and disease, patients may need to be screened for anti-Cas9 T cells and excluded from clinical trials. Targeting younger patients, who are less likely to have developed anti-Cas9 CTLs, or utilizing novel Cas9s with lower rates of pre-existing CTLs would minimize the number of patients that require exclusion. Tolerance induction could also be utilized in patients with pre-existing anti-Cas9 CTLs. AAV-directed liver expression has been shown to induce tolerance, even in the context of a pre-existing immune response^9,14^. Immune suppression such as with corticosteroids, which is often already utilized in AAV gene therapy, can also be used to minimize inflammation immediately following gene delivery and during initial expression both to prevent re-activation or initial development of anti-Cas9 T cells. While short-term immune suppression has proven tolerable in gene therapy trials, life-long suppression would be less tenable for many patients. Transient expression of Cas9 through self-destruction or nonviral delivery of mRNA or protein would shorten the time immune suppression would be required. Additionally, short-term (or muscle-restricted) expression would limit the impact of pro-inflammatory DNA damage signals, which could increase the chances of developing CTLs, kill or arrest Cas9-expressing cells^15^, or even theoretically lead to cancer^15^.

One final consideration that researchers must explore is how subsequent infections by *S. aureus*/*S. pyogenes* might impact anti-Cas9 immune responses. An active infection and the associated inflammation during a period of Cas9 expression could break tolerance, reverse anergy, and/or activate ignorant anti-Cas9 CTLs. Self-limited expression of Cas9 could shorten the period during which this is a relevant concern, but patients receiving gene transfer in a hospital setting, might have increased their risk of infection. Researchers working on therapies that will lead to long-term expression of Cas9 should especially attempt to address the issue in animal models of Cas9 immunity triggered by subsequent bacterial infections.

## Conclusions and future considerations

While Charlesworth et al.^5^ have demonstrated that concerns over the ability of the human immune system to mount anti-Cas9 responses are warranted^5^, various questions remain regarding the potential negative impact for gene therapy. An anti-Cas9 immune response leading to the killing of Cas9-expressing cells has yet to be seen in animal models following gene therapy with non-inflammatory vectors, such as AAV. It is thus unknown whether Cas9 expression in such a context—with or without pre-existing anti-Cas9 immunity—would lead to destruction of transduced cells. If CTLs do mediate killing following gene therapy, there are multiple strategies that researchers can utilize to minimize the development and impact of anti-Cas9 T cells. The gene editing field can find guidance from the gene therapy field, which has overcome anti-capsid and anti-transgene CTL responses by carefully considering vector, dose, target tissue, administration route, promoter, and immune suppression. CRISPR-Cas9 platforms that lead to short-term expression of Cas9 should also continue to be developed. While preclinical studies must address the issue of anti-Cas9 immune responses in immune-competent, large animal models, there is no reason to believe that any such challenges cannot be surmounted. As long as we proceed with caution, the future of gene editing is bright.
